# Nurses’ perception of uncertainty regarding suspected pain in people with dementia: A qualitative descriptive study

**DOI:** 10.1371/journal.pone.0300517

**Published:** 2024-04-04

**Authors:** Mohammad Rababa, Raghad Tawalbeh, Tala Abu-Zahra

**Affiliations:** 1 Department of Adult Health Nursing, Faculty of Nursing-Jordan University of Science and Technology, Irbid, Jordan; 2 University of Wisconsin-Milwaukee, Milwaukee, WI, United States of America; The University of Alabama, UNITED STATES

## Abstract

**Objectives:**

This study aims to qualitatively examine nurses’ perception of uncertainty regarding suspected pain in people with dementia (PWD).

**Design:**

The study utilized a qualitative descriptive design.

**Participants:**

The participants in this study were nurses with a minimum of six months of experience caring for PWD and currently working in a university hospital in Irbid, Jordan. Twenty-five participants were selected using convenience sampling from the selected hospital.

**Results:**

Four major themes and 12 subthemes relating to nurses’ perceptions of uncertainty regarding suspected pain in PWD emerged. The main themes were (a) the culture-bound nature of uncertainty regarding suspected pain in PWD, (b) dimensions of uncertainty regarding suspected pain in PWD, (c) indicators of uncertainty regarding suspected pain in PWD, and (d) assessment methods of uncertainty to suspected pain in PWD. Moreover, for each major theme, different subthemes were developed.

**Conclusions:**

It is crucial to address factors influencing -nurses’ uncertainty regarding suspected pain in PWD to improve pain assessment and management in PWD. Additionally, the study identified five indicators of uncertainty: complicated decision-making, knowledge deficit, bias, intuition, and misconceptions. Effective assessment methods, such as semi-structured interviews and simulated assessments, should be employed to evaluate uncertainty accurately. By addressing these issues and utilizing appropriate assessment approaches, healthcare professionals can enhance pain management for individuals with dementia.

## Introduction

Unrelieved pain is prevalent among people with dementia (PWD) due to difficulty assessing pain due to difficulties with self-expression, communication, and cognition [1]. Unrelieved pain in PWD is associated with irritability and physical mobility limitation, anorexia, social withdrawal, depression, delayed healing, insomnia, cognitive and functional decline, deteriorated quality of life, and increased morbidity and mortality rates [2]. Depending on the stage and progression of dementia, many PWD experience aphasia and cognitive deterioration, interfering with clearly articulating their needs to their nurses [3]. Given these pain assessment obstacles, nurses may either be uncertain regarding the presence of pain or misunderstand and misinterpret the behavioral pain indicators when the self-report of pain is more challenging. Thus, prompt and effective pain management is not delivered to PWD, resulting in persistent unrelieved pain [2].

Uncertainty regarding suspected pain in PWD is one of the significant barriers to prompt pain management [4]. For PWD, nurses mainly rely on assessing the atypical presentation of pain, including facial grimacing, guarding positions, meaningless movement, and aggressive behaviors [5]. Furthermore, this atypical presentation of pain is still unknown and unrecognized by most nurses caring for PWD. It is challenging for nurses who commonly use pain self-report tools to interpret the nonverbal indicators of pain manifested by PWD [5]. They may think these behaviors do not represent pain; they may be related to dementia, not pain [6].

As a result, nurses have deficient levels of certainty regarding suspected pain in PWD, and then they are uncertain of the imperative need of PWD for analgesics. Instead, nurses try other nonpharmacological interventions to manage those atypical behaviors related to pain [4]. These interventions include but are not limited to positioning, toileting, environment modification, minimizing noise, and outdoor activities. In addition, even if nurses administer analgesics, PWD may not receive an adequate dose of analgesics. Nurses do not prefer to take the risk of administering high doses of opioid analgesics to PWD if they are in need due to the fear of addiction and other misconceptions related to pain management in PWD [4]. Therefore, this hesitancy to administer opioid analgesics is associated with their uncertainty regarding suspected pain in PWD.

Consistently, Jordanian nurses feel uncertainty regarding suspected pain in PWD [2, 7]. In the context of Jordan, many reasons could contribute to this uncertainty, including knowledge deficit and malpractice regarding pain, as most Jordanian nurses have not been formally taught either at the graduate or undergraduate level about pain assessment and management in PWD [2, 7]. Furthermore, geriatric and dementia care in Jordan is lacking compared to Western countries. Jordanian healthcare settings do not provide their nursing staff with the required training or ongoing education in pain management in PWD [2]. Therefore, uncertainty regarding suspected pain is one of the major nurses’ perceived barriers to pain management in PWD [7].

Several studies suggest that the concept of uncertainty regarding suspected pain in PWD is culture-bound [3]. Compared to Western nurses, Jordanian nurses may conceptualize uncertainty regarding suspected pain in PWD differently. One study [3] suggested that the behaviors resulting from uncertainty may be culture-bound, finding that nurses were more comfortable reporting uncertainty regarding suspected pain in PWD, even if they were confident, as they always relate uncertainty to less accountability. Moreover, uncertainty may be typical among Jordanian nurses, especially when females are perceived as less confident than their Western counterparts [2, 8]. Noting that this novel research could be useful to help support better training for nurses on pain management for PLWD and improve pain management outcomes for PWD. To our best knowledge, no study has explored the conceptualization of uncertainty regarding suspected pain in PWD from the nurses’ perspectives. Therefore, this study aims to qualitatively examine nurses’ perception of uncertainty regarding suspected pain in PWD.

## Methods

### Design, setting, and sample

The study utilized a qualitative descriptive design [9] to examine Nurses’ perception of uncertainty regarding suspected pain in PWD. This research approach offers a high degree of flexibility in gathering and analyzing data, resulting in comprehensive and in-depth insights into the diverse perspectives of nurses [10]. Previous studies have successfully employed qualitative approaches to investigate nurses’ perceptions of various concepts related to clinical practice [11].

Thus, qualitative research was deemed suitable for addressing the gap in the literature concerning nurses’ perspectives on the concept of uncertainty in the context of pain assessment and management in PWD. The qualitative descriptive design enables researchers to describe and explore perceptions directly impacting educational settings. According to Bradshaw et al. [12], qualitative descriptive design is essential when examining participants’ perceptions of a poorly understood phenomenon.

Further, since limited knowledge exists regarding the factors influencing the conceptualization of uncertainty regarding suspected pain in PWD from the standpoint of Jordanian nurses, a descriptive qualitative design was chosen for the present study to elucidate and explore nurses’ perceptions on this topic. The study participants were nurses with at least one year of experience working in a university hospital in Irbid, Jordan. Twenty-five participants were selected using convenient sampling from the ICU, who were then interviewed to gather their perceptions.

### Data collection procedures

Prior to data collection, ethical approval was obtained from the institutional review board of the university (IRB 54/163/2023). A list of potential participants who met the eligibility criteria was acquired from the administrative office of the selected Hospital. The researchers approached all eligible individuals and requested their written consent to participate in the study.

The researchers conducted semi-structured face-to-face interviews with each participant individually. The interviews were conducted in Arabic and in a private and quiet setting. On average, the interviews lasted approximately 45 minutes. The interviews continued until data saturation was achieved, meaning that no further information was being obtained from the participants, at which point the interviews were concluded.

The participants were given four primary open-ended questions to elicit their perceptions and opinions regarding the concept of uncertainty regarding suspected pain in PWD. These questions aim to gather insights into the dimensions, indicators, and assessment methods of uncertainty regarding suspected pain in PWD. The researcher developed an interview guide (Table 1) to provide structure and guidance during the interviews. To ensure clarity of the questions, the interview guide was pilot tested with two ICU nurses, and any feedback they provided was considered for modifications. Any potential misunderstandings were addressed and clarified. Additionally, the interview guide included questions related to the participants’ sociodemographic and professional characteristics.

**Table 1:** Interview guide.

Interview guide
At the beginning of the interview, identify the participating nurses’ socio-demographic and professional characteristics details, including age, gender, marital status, year of experience as a registered nurse, and level of education.
(1) What were your experiences with caring for PWD?
(2) What were your perceptions of uncertainty regarding suspect pain in PWD?
(3) Compared to cognitively intact older adults, what were the barriers/facilitators to establishing certainty regarding suspected pain in PWD?
(4) How certain are you when assessing for pain in PWD?
(5) What are your attitudes, position, and decision about suspected pain in PWD?
(6) What factors influence your perception of uncertainty regarding suspected pain in PWD?
(7) What do you think are the best assessment methods available to capture the conceptual definition of uncertainty regarding suspected pain in PWD?
(8) What do you think are the components/indicators of the concept of uncertainty regarding suspected pain in PWD?

The interviews were recorded by the research assistant and transcribed verbatim in Arabic. The transcripts were coded to ensure the confidentiality and anonymity of the participants’ data. Aliases were assigned to each participant to protect their identities. Only the researcher had knowledge of the participants’ true identities.

Following transcription in Arabic, two nursing professors fluent in both Arabic and English independently translated the transcripts into English. The data, along with the transcripts, will be saved on a laptop secured with a password. The hard copies of the transcripts will be stored in a locked cabinet within a secure office that only the researcher can access.

### Data analysis

After thoroughly reading and re-reading the interview transcripts to gain a comprehensive understanding of their content, a thematic analysis approach [13] was employed. The choice of thematic analysis was driven by its flexibility and adaptability in exploring unexplored phenomena, identifying similarities and differences across the dataset, and comprehensively examining, organizing, analyzing, and reporting themes [13].

The interview transcripts were initially segmented into expressive units and labeled with relevant codes to represent their content. Descriptive codes were assigned to these units to aid in the identification of themes, keywords, and categories. The researcher and research assistant conducted comparisons between the different codes, then organized these codes into categories and themes. To ensure the rigor of the analysis process, two qualitative researchers who were not involved in the data collection phase will be consulted, and they independently reviewed the analysis. The researcher and research assistant thoroughly examined all the categories and further refined them by providing explanatory subcategories for the content within each category.

A subsequent meeting was held between the research team and the independent reviewers to discuss any data analysis and interpretation discrepancies. These discrepancies were resolved through internal discussions, with the involvement of a third experienced researcher for adjudication if necessary.

## Results

### Conceptualization of uncertainty regarding suspected pain in PWD

The analysis for the current study was developed with four major themes and 12 subthemes (Table 2) relating to nurses’ perceptions of uncertainty regarding suspected pain in PWD. The main themes were (a) the culture-bound nature of uncertainty regarding suspected pain in PWD, (b) dimensions of uncertainty regarding suspected pain in PWD, (c) indicators of uncertainty regarding suspected pain in PWD, and (d) assessment methods of uncertainty to suspected pain in PWD. Moreover, for each major theme, different subthemes were developed.

**Table 2:** Study themes and subthemes.

Themes	Subtheme
The culture-bound nature of uncertainty regarding suspected pain in PWD	
Dimensions of uncertainty regarding suspected pain in PWD	Nurse-related factors Patient-related factors System-related factors
Indicators of uncertainty regarding suspected pain in PWD	Complicated decision-making Knowledge deficit Bias Intuition Misconception
Assessment methods of uncertainty to suspected pain in PWD	Self-Assessment Interview assessment Simulated Assessment

### The culture-bound nature of uncertainty regarding suspected pain in PWD

Most nurses found uncertainty regarding suspected pain in PWD a complex concept to operationalize. It describes the degree to which nurses feel they can be certain that pain is likely present. Essential characteristics of uncertainty regarding suspected pain are that it is strongly culture-bond. One nurse stated when asked, *“What do you think about uncertainty regarding suspected pain in PWD*?*”*: *“That*, *of course*, *depends on the cultural context where the nurse practices nursing*, *especially if she is a female”* (N4). Another nurse stated: “*Uncertainty regarding suspected pain in PW is different from one nurse to another”* (N7).

The nurses in the current study conceptualized uncertainty regarding suspected pain in the context of Jordanian culture. The nurses reported that their administrators severely restricted their certainty regarding suspected pain in PWD. One nurse said, *“our manager predetermined how we should assess pain in PWD regardless of my level of certainty regarding suspected pain”* (N13). Nurses were more comfortable when reporting uncertainty regarding suspected pain in their patients, as it implies less accountability. A nurse stated, *“to keep me out of trouble*, *I always state uncertainty when assessing pain in PWD”* (N9). This perception was reported more frequently among female nurses than male ones because women seem less confident than men in Middle Eastern culture. Consequently, nurses in the current study were frequently blamed by their managers for their decisions related to pain management in PWD, especially when they were wrong. One nurse reported, *“no matter what the outcome of my decision about pain in my patient*, *my manager always blames me anyway”* (N15). This “blame culture” impedes nurses’ interest in making autonomous decisions due to the fear of being criticized by their managers. The nurses in the current study were uncertain about making decisions related to pain management if they thought their boss would blame them for these decisions. A nurse stated, *“my manager is pressuring me a lot and they blame me for the wrong decisions*. *This makes me unconfident about my ability to assess and manage pain in PWD”* (N22).

### Dimensions of uncertainty regarding suspected pain in PWD

#### Nurse-related factors

Nurses reported different nurses-related factors contributing to uncertainty regarding pain in PWD. These factors include high prevalence of myths related to pain management among nurses caring for PWD. Many myths related to pain management contribute to uncertainty regarding suspected pain in PWD, including it is difficult to perform accurate pain assessment among PWD because pain is self-reported and opioid usage in PWD is useless. A nurse reported, “I feel frustrated when I cannot figure out what I should do for my suffering patients with dementia” (N20). Another exclaimed, *“The most difficult thing you imagine doing is the administration of analgesics to PWD; I do not feel that they gonna benefit them*. *Actually*, *I think when we do so*, *we put them at great risk for addiction”* (N21). One nurse stated, *“PWD cannot tolerate even minimal doses of opioid analgesics because we don’t have any clue that they are actually in pain”* (N3). Another nurse said, *“Even if they are in pain*, *as long as they don’t complain*, *no need to prescribe analgesics for them*. *Let’s say that we give them analgesics for their pain; how come we can assess their response to analgesics*!*”* (N6).

#### Patient-related factors

As for patient-related factors, nurses reported patients’ inability to communicate verbally, misunderstanding of the behavioral indicators of pain when self-report is more difficult, and inconsistency in these behavioral and physiological indicators of pain from one patient to another. One nurse reported that *“I am not sure about self-report of pain from a PWD with aphasia; however*, *I am very sure about suspected pain in a PWD who is still able to self-report*.*”* (N8). Another nurse stated, *“I will never be certain that a PWD has pain until they said that; we are not taught in the school how to predict that someone is in pain with no overt signs*!*”* (N10). Another nurse stated, *“Even when trying to understand the odd behaviors acted by a PWD*, *these behaviors seem unreliable indicators of pain as they are inconsistent from one PWD to another*.*”* (N12)

#### System-related factors

As system-related factors, nurses mentioned the shortage of palliative care specialists, an inadequate nurse-to-patient ratio, and intensive workloads. A nurse stated, *“I have so many commitments …*. *I work with many patients; therefore*, *I have no time to do additional assessments when needed to compromise my uncertainty regarding suspected pain in PWD”* (N3). Another nurse said, *“We don’t employ any evidence-based protocol/ guideline for pain assessment and management tailored specifically for PWD”* (N1). Also, nurses reported that despite the availability of different tools and methods for pain assessment, the inconsistency in using these tools might introduce uncertainties in pain assessment among PWD. One nurse stated, *“We don’t even utilize any measurement tools designed specifically for pain in nonverbal PWD”* (N4). Another reported, *“We only use a numerical pain scale for all patients*, *including those who are unable to talk*!*”* (N7).

Moreover, a lack of knowledge and poor nursing education related to pain assessment and management in PWD were system-related factors. One nurse reported, “We have not received formal education about dementia care or pain management in PWD in undergraduate nursing studies” (N11). Another said, “Moreover, nursing educators underestimate the importance of geriatric care or gerontology; these topics are actually integrated into other nursing topics, and the educators only refer to them as self-readings” (N19). Nurses reported knowledge deficits regarding the recommended routes and doses of opioids and an inability to assess and reassess the patient’s condition after the administration of opioids.

### Operationalization of uncertainty regarding suspected pain into measurable indicators

#### Indicators of uncertainty regarding suspected pain in PWD

Five relevant, measurable indicators were found to assess uncertainty regarding suspected pain in PWD: complicated decision-making, knowledge deficit, bias, intuition, and misconception. These indicators are outlined below.

#### Complicated decision making

Most participating nurses reported that people uncertain regarding suspected pain in PWD had complicated decision-making during pain assessment and management in PWD. They described the decision-making process as a multidimensional systematic process requiring the nurses to understand all relevant factors comprehensively. One nurse said,” *It’s tough to decide whether they are in pain because they cannot tell you that*. *Even if I think they have pain*, *I actually don’t know… I am just guessing”* (N8). Instead, most nurses highlighted that they were stuck in a trial-and-error approach which failed to compromise their uncertainty regarding suspect pain in PWD. A nurse said, *“Well*, *with PWD*, *I think I must narrow my decision options*. *Maybe I have to toilet them*, *or I must feed them*, *or I must provide a message*. *I think some PWD are still in pain even if given a pain pill because they need to go to the toilet or have food*. *So*, *it’s a matter of feeding or toileting …*. *It seems kind of a hit-and-miss”* (N8). In this approach, nurses reported administering paracetamol, observing PWD’s responses to the medicine, administering nonpharmacological interventions, and just looking for disappearing atypical behaviors related to pain.

#### Knowledge deficit

Nurses reported a lack of knowledge deficits regarding the atypical presentation of diseases in older adults who cannot self-report pain. They acknowledge that this knowledge deficit causes their uncertainty regarding suspected pain in PWD. One nurse said that *“I think that quiet PWD sometimes is forgotten …*. *I think for those striking out; it is easier to figure out maybe they are in pain… I think we should deal with many things when caring for those suffering PWD*. *We should assess their unmet needs*, *such as toileting*, *feeding*, *anxiety*, *etc*.*”* (N10). Another nurse said, *“It’s much easier to establish certainty of pain when caring for verbal patients; you only need to ask them to rate their pain… In the case of PWD*, *I don’t even ask because I know they wouldn’t understand it anyway… I become frustrated and don’t know what I should do”* (N15). They reported misunderstanding the available protocols for assessing pain in PWD. One nurse stated, “*No matter how I care for a PWD*, *he/she will still be in pain*. *I have just gone through the motions of assessment for pain in a PWD; I don’t know how to assess pain in PWD with confidence*” (N14).

#### Misconceptions

Nurses reported that those uncertain about pain in PWD always have several misconceptions about pain assessment and management in PWD. Some nurses think that pain is a part of dementia. For example, one nurse stated, “*Most PWD have many coexisting health problems*, *such as osteoarthritis and chronic constipation*, *and They have frail bodies*, *so they must complain of pain*” … *we try our best to manage their pain*. *However*, *it is typical for such a group of patients to have pain*” (N9). Some participating nurses seem to misunderstand the process of dementia and how it relates to the problem of uncertainty regarding pain in PWD. A nurse said, “*As long as a person has dementia*, *they should complain from pain”* (N8). Another nurse said, *“Actually*, *PWD are less sensitive to pain “*(N11). Also, another nurse reported, *“As long as PWD report no pain*, *they don’t have pain”* (N16). Also, nurses reported that they do not try their best to establish certainty regarding pain in PWD because they underestimate the use of analgesics for PWD. One nurse stated, *“PWD don’t get benefits from using analgesics”* (N19). Another nurse exclaimed, *“PWD should not be prescribed opioid analgesics as they will be addicted to them”* (N21).

#### Intuition

Most nurses stated that they rely on intuition when assessing pain in PWD. They have difficulty with the complexity of pain assessment in PWD; therefore, they try to guess whether a PWD has pain. Nurses caring for PWD reported that they prefer a trial-and-error approach when managing pain as they do not have adequate training and support for this complex decision-making process. Due to the complexity of pain management in PWD, nurses must understand the complicated links between all variables related to pain- management in PWD. However, nurses in the current study reported poor education and training on how systematically and critically unfold the multicomponent structure of pain concept in PWD. Instead, they reported overreliance on the guessing approach. For example, nurses reported that “*they just feel that something wrong inside their patients is wrong as they are not being themselves*” (N17). Another nurse said,” *Some PWD can’t speak anymore about what they are feeling*, *so I’m relying more on my inner feelings or perception of what I think they may feel”* (N8). Another nurse consistently reported, “*I rely on my intuition when assessing for pain in a PWD because I am not sure if the odd behavior exhibited by my patient manifests pain*” (N9).

#### Bias

Many nurses reported being quite sensitive to pain expression when assessing for it. Nurses stated that they underestimate the experience of pain by PWD, which contributes to uncertainty regarding suspected pain in PWD. One nurse stated, “*Because we nurses have many years of clinical experience caring for PWD*, *we are overexposed to different atypical manifestations of pain”* (N10). Nurses reported referring to their own perception and experience of pain to clearly understand their patient’s pain nature, which would introduce a rater bias.

#### Assessment methods of uncertainty to suspected pain in PWD

Quantifying the degree to which nurses feel they can be certain that pain is likely present is considered a huge challenge. As one nurse indicated, *“It is easy to decide that someone is uncertain regarding suspected pain*, *but when it comes to assessing the degree of uncertainty regarding suspected pain in PWD*, *we are stuck in a very long complicated process”* (N4). After we explored how best to assess the indicators of uncertainty regarding suspected pain in PWD, three methods emerged: self-assessment, assessment interviews, and simulated assessment. Although participants disagreed that a single method could best assess uncertainty regarding suspected pain in PWD, most participants found that several methods should assess it.

Self-assessment alone was not considered a reliable measure. One nurse stated, “*nurses with insufficient knowledge and inadequate clinical training may have difficulty determining their own capabilities”* (N1). Nurses also pointed out that self-assessment of cognitive skills, capabilities, and perceptions might need to be completed with further assessment, as employed by semi-structured interviews. Although most nurses considered a semi-structured interview an important assessment method, especially when combined with additional methods, nurses found such interviews unreliable. They believed that the method was too naïve; as one nurse highlighted, “*The interviews are so simple*, *and I don’t think that would be effective”* (N5); they stated that the interviews should also have “examples of daily experiences, details from other healthcare professionals, and double-checking for discrepancies,” as well as “characteristics of the patients and the cultural context” (N6).

The participants highlighted that a simulated assessment that utilizes simulated case scenarios could be a valid assessment method that fills a gap in understanding nurses’ conceptualization of uncertainty regarding suspected pain in PWD. Meanwhile, the nurses highlighted that the simulated assessment is a structured assessment method that provides a step-by-step understanding by analyzing and synthesizing nurses’ knowledge of uncertainty regarding suspected pain. One nurse stated, “*Simulated case scenarios on PWD helped me systematize my knowledge related to establishing certainty regarding suspected pain in PWD”* (N8). This simulated setting makes it possible to test and observe indicators of uncertainty regarding suspected pain in PWD. As a nurse stated, *“Observe if I make mistakes or decide with a high level of certainty regarding the presence of pain in my patients”* (N9).

## Discussion

This is the first qualitative study to examine nurses’ perceptions of uncertainty regarding suspected pain in PWD.

### Characteristics of uncertainty regarding suspected pain in PWD

The findings indicate that uncertainty regarding suspected pain is a complex concept for nurses to operationalize. Cultural factors, particularly in Middle Eastern culture, strongly influence nurses’ perception of uncertainty [2]. Due to cultural expectations, female nurses tend to express more uncertainty than male nurses. Additionally, the study revealed a blame culture within the nursing environment, where nurses felt hesitant to make autonomous decisions regarding pain management in PWD for fear of being criticized by their managers [8]. Thus, addressing cultural factors and the blame culture is crucial for improving pain assessment and management in PWD.

### Dimensions of uncertainty regarding suspected pain

*Nurse-related factors*. The current study found that key dimensions of uncertainty regarding suspected pain in PWD are not only related to nurses themselves but also to the patient and system-related factors. The nurse-related factors identified in this study shed light on the contributors to uncertainty regarding pain assessment and management in PWD. The study also reveals that nurses’ inadequate pain assessment and knowledge deficits are significant barriers to achieving optimal pain management for PWD [14, 15]. Nurses reported a lack of understanding regarding recommended routes and doses of opioids and challenges in assessing and reassessing the patient’s condition after opioid administration [5, 16]. Moreover, various myths related to pain management add to the uncertainty surrounding suspected pain in PWD. These myths include beliefs that pain assessment is challenging in PWD because it relies on self-reporting, concerns about the potential risks of opioid usage, and the assumption that PWD does not require analgesics unless they explicitly complain of pain [1].

The nurses’ expressions of frustration and uncertainty reflect their struggle to determine the appropriate course of action for their patients with dementia. Some nurses doubted the effectiveness of administering analgesics, questioning whether PWD would truly benefit or be at risk for addiction. There were concerns about PWD’s tolerance for even minimal doses of opioid analgesics and the difficulty in assessing their response to pain management intervention [5, 16].

*System-related factors*. Nurses discussed system-related factors contributing to pain assessment uncertainty for PWD. These factors include a shortage of palliative care specialists, inadequate nurse-to-patient ratios, and heavy workloads. In addition, nurses mentioned the availability of different pain assessment tools but expressed concerns about the inconsistency in their use. They reported a lack of evidence-based protocols or guidelines specifically tailored for pain assessment and management in PWD. Additionally, nurses mentioned the absence of measurement tools designed specifically for pain assessment in nonverbal PWD, relying instead on a numerical pain scale used for all patients regardless of their verbal ability.

Furthermore, the findings highlight a lack of knowledge and poor attitudes among nurses as significant factors. Also, nurses reported a lack of formal education on dementia care and pain management specific to PWD during their undergraduate nursing studies. The nurses expressed frustration and dissatisfaction with the limited coverage and importance assigned to geriatric care and gerontology by nursing educators, often relegating them to self-readings rather than integral components of the curriculum.

These nurse-related and system-related factors highlight nurses’ challenges in accurately assessing and managing pain in PWD. Difficulties interpreting behavioral indicators and lacking specialized assessment tools contribute to uncertainty [17–19]. Moreover, system-related issues such as resource limitations and workload pressures exacerbate the problem [5, 20]. Addressing these factors through education, improving resource allocation, and implementing evidence-based protocols specific to PWD can help reduce uncertainty and enhance pain management for this vulnerable population [4–6].

### The operationalization of uncertainty regarding suspected pain into measurable

*Indicators of Uncertainty regarding suspected pain*. Indicators are essential for adequate pain assessment and management in PWD [18, 21]. This study identified five relevant indicators: complicated decision-making, knowledge deficit, bias, intuition, and misconception. Understanding and addressing these indicators can improve pain assessment and management in PWD [2, 22].

The first indicator, complicated decision-making, emerged as a significant factor contributing to uncertainty regarding suspected pain in PWD [4, 23, 24]. Nurses described the decision-making process as challenging and multidimensional, requiring a comprehensive understanding of various factors. For some persons living with dementia, it is difficult to express their needs/communicate their level of pain, which may lead nurses to rely on guesswork and a trail-and-error approach [25]. This highlights the need for more structured and systematic approaches to pain assessment in PWD, considering individualized factors such as toileting, feeding, and nonpharmacological interventions [4, 23, 24].

The second indicator, knowledge deficit, reflects the lack of understanding among nurses regarding the atypical presentation of diseases in older adults and the unique challenges faced when assessing pain in PWD [26]. Nurses acknowledged their limited knowledge and reported difficulty in assessing pain in PWD due to their inability to self-report clearly [14, 27, 28]. This knowledge gap can contribute to uncertainty and frustration among nurses, leading to suboptimal pain management. To address this indicator, educational interventions should enhance nurses’ knowledge about pain assessment and management tailored to PWD [29, 30].

Whereas bias, intuition, and misconception emerged as additional indicators of uncertainty regarding suspected pain in PWD [3, 4, 18]. Nurses reported biases, particularly toward more vocal or agitated PWD, which may lead to overlooking pain in quieter individuals. While sometimes valuable, intuition should be supported by evidence-based assessment strategies to minimize uncertainty. Misconceptions about the effectiveness of pain assessment protocols in PWD were also reported, emphasizing the need for clear guidelines and training on appropriate pain assessment tools and techniques.

Thus, operationalizing uncertainty regarding suspected pain in PWD into measurable indicators is crucial for effective pain assessment and management [31]. The identified indicators, complicated decision-making, knowledge deficit, bias, intuition, and misconceptions highlight healthcare professionals’ challenges. Combining assessment methods such as self-assessment, assessment interviews, and simulated assessment can provide a comprehensive understanding of nurses’ uncertainty and aid in developing targeted interventions and training programs to improve pain management in individuals with dementia.

*Assessment methods of uncertainty to suspected pain in PWD*. Assessing uncertainty regarding suspected pain in individuals with dementia poses a significant challenge for healthcare professionals [3, 17]. Self-assessment alone was deemed unreliable due to potential knowledge deficits and inadequate clinical training [4]. Nurses recognized the importance of incorporating additional assessment methods, such as semi-structured interviews, to validate their self-assessment and gain a more comprehensive understanding of their capabilities [32]. However, concerns were raised about the simplicity of the interview method, suggesting the need for enhanced interview techniques that include real-life examples, input from other healthcare professionals, and considerations of patient characteristics and cultural context.

A promising assessment method highlighted by participants was simulated assessment using case scenarios. This structured approach allowed nurses to systematically analyze and synthesize their knowledge of uncertainty regarding suspected pain in individuals with dementia. Simulated case scenarios provided valuable opportunities for nurses to test and observe the indicators of uncertainty, enabling them to assess their decision-making process and identify areas where mistakes might occur or where a high level of certainty could be achieved in determining the presence of pain. By employing multiple assessment methods, healthcare professionals can obtain a comprehensive evaluation of uncertainty regarding suspected pain in individuals with dementia, facilitating the development of effective interventions and training programs in pain management.

## Conclusion

This qualitative study focused on nurses’ perception of uncertainty regarding suspected pain in PWD. The findings revealed various factors contributing to uncertainty, including cultural influences, a blame culture, lack of knowledge and education, system-related challenges, and inadequate assessment methods. It is crucial to address these factors to improve pain assessment and management in PWD. Additionally, the study identified five indicators of uncertainty: complicated decision-making, knowledge deficit, bias, intuition, and misconceptions. Effective assessment methods, such as semi-structured interviews and simulated assessments, should be employed to evaluate uncertainty accurately. By addressing these issues and utilizing appropriate assessment approaches, healthcare professionals can enhance pain management for individuals with dementia.

## Recommendations

The study examined nurses’ perceptions of uncertainty regarding suspected pain in PWD and identified two major themes: "Characteristics of uncertainty" and "Dimensions of uncertainty." Nurses reported that cultural influences, knowledge deficits, biases, and reliance on intuition contribute to uncertainty. Patient-related factors included communication difficulties and inconsistent pain indicators, while system-related factors encompassed resource shortages and a lack of specific assessment tools. Indicators of uncertainty included complicated decision-making, knowledge deficit, bias, intuition, and misconceptions. The study suggested self-assessment, assessment interviews, and simulated assessment to measure uncertainty. Overall, the findings emphasize the need for improved education, training, and evidence-based protocols to enhance pain management in PWD.
